# Physical Activity Level Using Doubly-Labeled Water in Relation to Body Composition and Physical Fitness in Preschoolers

**DOI:** 10.3390/medicina55010002

**Published:** 2018-12-27

**Authors:** Marja H. Leppänen, Pontus Henriksson, Hanna Henriksson, Christine Delisle Nyström, Francisco Jesus Llorente-Cantarero, Marie Löf

**Affiliations:** 1 Faculty of Sport and Health Sciences, University of Jyvaskyla, FI-40014 Jyvaskyla, Finland; 2 Department of Biosciences and Nutrition, Karolinska Institutet, SE-141 83 Huddinge, Sweden; pontus.henriksson@ki.se (P.H.); christine.delisle.nystrom@ki.se (C.D.N.); marie.lof@ki.se (M.L.); 3 Department of Medical and Health Sciences, Linköping University, 581 83 Linköping, Sweden; hannahenriksson77@hotmail.se; 4 Healthy Active Living and Obesity (HALO) Research Group, Children’s Hospital of Eastern Ontario Research Institute, Ottawa, ON K1H 8L1, Canada; 5 Department of Physical Education and Sport, Faculty of Education, University of Sevilla c/ Pirotecnia, s/n, 41013 Sevilla, Spain; llorentefj@yahoo.es; 6 Instituto Maimónides de Investigación Biomédica de Córdoba (IMIBIC), University of Cordoba, CIBERObn, Avda Menéndez Pidal s/n, 14004 Cordoba, Spain

**Keywords:** energy expenditure, obesity, cardiorespiratory fitness, muscular strength, children

## Abstract

*Background and objectives*: There is a lack of studies investigating associations of physical activity level (PAL) and activity energy expenditure (AEE) using the doubly-labeled water (DLW) method with body composition and physical fitness in young children. Thus, we aimed to examine cross-sectional associations of PAL and AEE with body composition indices and physical fitness components in Swedish preschool children. *Materials and methods:* PAL was calculated as total energy expenditure measured using DLW divided by the predicted basal metabolic rate in 40 children aged 5.5 (standard deviation 0.2) years. AEE was calculated as total energy expenditure minus basal metabolic rate and the thermic effect of food, and divided by fat-free mass. Body composition was assessed using the 3-component model by combining measurements based on isotope dilution and air-displacement plethysmography. Physical fitness (muscular strength, motor fitness, and cardiorespiratory fitness) was evaluated using the PREFIT test battery. Multiple linear regression models were conducted. *Results:* PAL and AEE were negatively associated with body mass index, percent body fat, and fat mass index (PAL: standardized β −0.35, −0.41, and −0.45, all *p* < 0.036; AEE: standardized β −0.44, −0.44, and −0.47, all *p* < 0.006, respectively). Furthermore, PAL and AEE were positively associated with the standing long jump test (PAL: standardized β 0.37, *p* = 0.017; AEE: standardized β 0.38, *p* = 0.014). There were no statistically significant associations found regarding PAL or AEE with fat-free mass index or any other physical fitness test. *Conclusions:* Greater PAL and AEE at the age 5.5 were significantly associated with body fatness and improved lower-body muscular strength. Therefore, increasing physical activity, and thus energy expenditure, at young ages may be beneficial for preventing overweight/obesity. However, further studies with larger sample sizes are needed to confirm the results.

## 1. Introduction

Overweight/obesity in childhood is one of the most serious public health challenges of the 21st century [1] and causes multiple adverse health consequences [2,3]. Worldwide, over 42 million children under 5 years of age are estimated to be overweight/obese [1]. In Sweden, the prevalence of children (aged 2–10 years) with overweight and obesity is ~10–15% [4]. These facts are concerning as overweight has been found to track from childhood to adolescence [5,6,7]. Therefore, interventions in young children are required, to attempt to prevent the development of overweight/obesity, and consequently, lower the risk of noncommunicable diseases [1,2].

Physical activity (PA) affects positively on energy balance, and thus, it has been considered an important factor in counteracting childhood obesity. The doubly-labeled water (DLW) method is the gold standard in measuring total energy expenditure (TEE) during free-living conditions [8]. When applied correctly, the DLW method can produce estimates of TEE with an accuracy of 1–3% and a precision of 2–8% [9]. Moreover, the use of the multipoint protocol (i.e., several postdose samples spread out during the 10–14 day turnover period) instead of the two-point protocol (i.e., postdose samples on the first day of dosing when isotopic equilibrium has been established and in the end of the turn-over period) has been suggested to be used in association studies when maximum precision is desired [10]. Together with basal metabolic rate (BMR), TEE provides reference estimates of activity energy expenditure (AEE) and physical activity level (PAL), which are essential in identifying associations between energy expenditure in response to PA and body composition.

Considering the impact of PA on energy expenditure it is relevant to examine the associations of PAL and AEE with body fatness in childhood. The few conducted studies in school-aged children have reported that PAL is inversely correlated with fat mass [11,12,13]. However, obesity in very young children has been associated with later disease [1,2] and considering that obesity at a young age tracks into later life [5,6,7], it may be of great importance to also consider the relevance of PA to prevent obesity already at the preschool age. However, to date, very few studies [14,15,16,17] have examined the associations of PAL and AEE with body composition in young children and these studies have reported somewhat contradictory results. For instance, Salbe et al. [14] and Eriksson et al. [15] found inverse associations for PAL or AEE with fat mass, although two other studies did not report such associations [16,17]. These differences may be due to differences in methods, statistical analysis, and characteristics of study participants. Importantly, the few available studies in preschool-aged children warranted further investigation to increase the knowledge of early PA on body composition indices. This is of importance considering the negative influence of obesity already in the preschool age [1,3,5,6].

Furthermore, physical fitness has been found to be an important health indicator [18,19,20,21]. For example, Ortega et al. [20] found an inverse relationship between physical fitness levels and obesity in children and adolescents and corresponding associations between fitness and fat mass has also been reported in preschool-aged children [22,23].

Therefore, the purpose of this study was to examine the associations between PAL and AEE assessed using the DLW method with body composition indices and physical fitness measures in healthy Swedish children aged 5.5 years. Our hypothesis was that a higher PAL and AEE are associated with favorable body composition and improved physical fitness.

## 2. Materials and Methods

### 2.1. Participants and Study Design

During the spring of 2015, parents from the MINISTOP trial (n = 315) (clinicaltrials.gov: NCT02021786) were asked to participate with their child in a validation study for the dietary and physical activity measures at the 12-month follow-up measurement, when the child was 5.5 years of age [24,25]. MINISTOP’s inclusion criteria were parents having a healthy 4-year-old child, having the possibility to have baseline measurements assessed at 4.5 years of age, and for at least one parent to be able to speak/read Swedish sufficiently well. Children with neurological or endocrine diseases, or if their parents suffered from a physical or psychological disease, were excluded from the study. A total of 45 parent child dyads were consecutively asked to participate until 40 agreed to do so (Figure S1). All of the children who agreed to participate in the validation study completed all three measurements within the MINISTOP trial. The children and their parents were comparable to the entire trial in terms of parental age, education, and children’s body mass index (BMI). The recruitment and study protocol for this validation study has been provided elsewhere [24,25]. In brief, anthropometric measures (body weight and height) were assessed using standardized procedures [24,25]. Body volume was measured in the BodPod (COSMED USA Inc., Concord, CA, USA) utilizing the relationship between pressure and volume [26,27] and then converted to fat mass and fat free mass. Physical fitness was assessed using the PREFIT test battery (FITness testing in PREschool children) [28]. All measurements occurred at the University Hospital in Linköping. The child then received a dose of DLW in order to measure the child’s TEE and total body water. The parents were provided with instructions and equipment in order to collect five post dose urine samples during the subsequent 14 days [24]. Informed verbal consent, witnessed and formally recorded, was obtained from the parents. The Research Ethics Committee, Stockholm, Sweden (2013/1607-31/5; 2013/2250-32) approved all procedures described.

### 2.2. Energy Expenditure in Response to Physical Activity

The deuterium and oxygen-18 enrichments of dose, pre and post dose urine samples (day 1, 5, 7, 10 and 14) were analyzed using a Finnigan MAT Delta Plus Isotope-Ratio Mass Spectrometer (ThermoFinnigan, Gothenburg, Sweden). Thereafter, total body water and TEE were calculated in accordance with Slinde [29]. PAL was calculated as TEE divided by the BMR which was computed using prediction equations based on weight [30]. AEE was calculated as TEE minus BMR and the thermic effect of food, which was assumed to be equal to 10% of TEE.

### 2.3. Fat and Fat-Free Mass

Fat mass was calculated using the 3-component model [31] utilizing measurements of body volume, total body water, and body weight. The following equation was used. Fat mass (kg) = ((2.220 × body volume) − (0.764 × total body water)) − (1.465 × body weight) [32].

Fat-free mass (kg) was calculated as body weight minus fat mass, while percent body fat (BF%) was calculated as fat mass (kg)/body weight × 100. Moreover, we included the body composition indices BMI, fat mass index (FMI), and fat-free mass index (FFMI). BMI was calculated as body weight (kg) divided by height^2^ (m), FMI as fat mass (kg) divided by height^2^ (m), and FFMI as fat-free mass (kg) divided by height^2^ (m).

### 2.4. Physical Fitness

Physical fitness was assessed using the PREFIT fitness test battery that has been suggested to use in preschool-aged children [28]. Cardiorespiratory fitness was measured using the 20-m shuttle run test. Briefly, the child ran between two lines 20-m apart with a trained research staff starting at 8.5 km/h and increasing 0.5 km/h/min. Upper-body muscular strength was assessed using the handgrip strength test with a dynamometer (TKK 5001, Grip-A, Takei, Tokyo, Japan). Lower-body muscular strength was assessed using the standing long jump test. Motor fitness was measured using the 4 × 10-m shuttle run test where the child ran between two lines 10-m apart four times as fast as they could. All of these, except the 20-m shuttle run test, were applied twice. For handgrip strength, the best attempts for the right and left hand (2 attempts per hand) were averaged and used in the analyses. With regard to the 4 × 10-m shuttle run and the standing long jump tests, the best value of the two attempts were included. The physical fitness measurements have been previously reported in more detail [23,27].

### 2.5. Statistical Methods

We examined if main study variables were normally distributed using the Shapiro–Wilk test. Physical activity data (i.e., PAL and AEE per kg fat-free mass), physical fitness data, and BF% were normally distributed, whereas BMI, FMI, and FFMI were not. Therefore, values are presented as means ±SDs, or medians (ranges) based on their distributions. Linear regression analysis was applied to examine the cross-sectional associations of (1) PAL and (2) AEE with body composition indices and physical fitness. Since fat-free mass has been found to be an important predictor of energy expenditure [33], we divided AEE (in kJ) with fat-free mass (in kg). This validation trial was powered so that a sample size of 40 children could detect standardized regression coefficients of 0.42 with 80% power (α = 0.05 and two-tailed).

We also divided AEE (in kJ) by body weight (kg), and the results were consistent with the models for AEE adjusted by fat-free mass (data not shown). All models were adjusted for the child’s age and sex. Additionally, models were adjusted for maternal education (university degree versus no university degree). Maternal education did not influence the results and therefore it was not included in the final models. In sensitivity analyses, for the models investigating the associations of PAL with BMI, BF%, and FMI they were additionally adjusted for fat-free mass (in kg) in order to examine its influence on the associations. In these models, AEE was used without adjustment for fat-free mass. In all of the models, we added an interaction term to the adjusted regression models in order to examine whether the associations differed between boys and girls. However, there was little evidence for sex-interactions between PAL or AEE and body composition or physical fitness, and therefore, the results were presented for boys and girls together.

The statistical software used was SPSS Statistics 24 (IBM, Armonk, NY, USA) and all tests were two-sided with *p* < 0.05 as the predefined significance level.

## 3. Results

The participating children’s mothers (n = 40) had an average age of (years) 36 ± 4.2, average height (cm) of 1.67 ± 0.06, average weight (kg) of 68 ± 11.4, average BMI (kg/m2) of 24.3 ± 4.0, and 72.5% of them (n = 29) had completed a university degree. The background information of the children (22 boys and 18 girls) is presented in Table 1. The children covered a wide range for body composition, TEE, AEE, PAL, and physical fitness.

Table 2 shows the associations between PAL and AEE with body composition measurements. After adjusting for sex and age, a higher PAL was significantly inversely associated with a lower BMI, BF%, and FMI (standardized β −0.35, −0.41, and −0.45, all *p* < 0.036). Similarly, a higher AEE was associated with a lower BMI, BF%, and FMI (standardized β −0.44, −0.44 and −0.47, all *p* < 0.006).

In the sensitivity analyses, when we also adjusted the models with PAL as a dependent variable for fat-free mass, the significant associations of PAL with BMI, BF%, and FMI became stronger (standardized β −0.42, −0.47, and −0.48, all *p* < 0.001).

With regards to physical fitness, a 0.1 higher PAL was associated with a 5.28 cm better score for the standing long jump test after adjusting for confounders (standardized β 0.37, *p* = 0.017) (Table 3). Furthermore, a higher AEE was associated with a better score for the standing long jump test (standardized β 0.38, *p* = 0.014). There were no statistically significant associations found between PAL or AEE with any other physical fitness tests or FFMI (all *p* > 0.05).

## 4. Discussion

The main results of this study are that a higher PAL and AEE were associated with a lower BMI, BF%, and FMI as well as with better lower-body muscular strength. This is of great importance, since a healthier body composition in childhood has been found to be associated with a healthier cardiovascular profile later in life and with a lower risk of death [19]. Furthermore, the positive association between PAL and AEE with lower-body muscular strength is important, as muscular strength has been found to be positively connected to perceived health status and life satisfaction in children [21]. Additionally, improvement in muscular strength from childhood to later in life has been found to be negatively related to changes in overall adiposity [19] as well as positively related with skeletal health [18].

Previous studies have also reported significant associations between a higher PAL and AEE with a lower BF%, fat mass, and BMI [12] in children as well as between a higher AEE and a lower fat mass [14]. However, in a cross-sectional study, Butte et al. [16] found no relationship between PAL and body composition indices in 4.5-year-olds. Other studies in younger [15] and older [11] children have found that PAL has been negatively associated with fat mass but not with fat-free mass. Moreover, Butte et al. [16] found significant associations between AEE and BMI, but not with BF% or fat mass. It is important to note that fat-free mass was not taken into account in their analyses, and since fat-free mass has been found to have an important role in energy expenditure [33], this may be a possible reason for the observed differences between the two studies. Rennie et al. [13] adjusted their analyses for fat-free mass, and they found significant relationships between PAL and AEE with FMI. Similar to our study, the relationships were weaker without adjusting for fat-free mass. However, despite adjusting for fat-free mass, they observed no association between AEE and BMI [9]. This may be due to differences in sample sizes (100 children vs. 40 children) and study populations (e.g., regarding activity patterns). However, these findings further emphasize the need for proper adjustment for body size when investigating the association between PAL and AEE with body composition indices in children.

We did not observe any significant associations between PAL and FFMI, which is in line with a previous study [16], but contradictory to one another [13]. The inconsistent results may be partly explained by the differences in the utilized methodologies as mentioned above, or it could also be due to the fact that PAL reflects overall PA and not a specific intensity level. High-intensity PA has been found to be positively related to FFMI [27], and thus, it is possible that the children in our study had less high-intensity PA compared to the children in the other study [13]. Since heart rate has been found to influence energy expenditure [34], having more time spent in low-intensity PA may result in similar energy expenditure levels as having less time spent in high-intensity PA. Although the influence on body composition may be different. This is important since children differ in terms of how they perform PA, with some children preferring low-intensity PA while the others are more interested in high-intensity PA. 

PAL and AEE were both positively associated with better lower-body muscular strength, but not with other components of physical fitness. To our knowledge, there are no previous studies examining the associations between PAL or AEE with physical fitness in preschoolers. Thus, comparison to other studies is not possible. However, our findings are reasonable, since PA in young children may be more focused on maintaining and improving the lower-body muscles (i.e., running, jumping, and cycling). In our previous study [27], we observed a positive association of high-intensity PA assessed using accelerometry with cardiorespiratory fitness and motor fitness. The lack of significant associations in this study may be due to the fact that the children had less high-intensity PA as discussed earlier, as the accelerometer data showed that they only performed on average 7 min of vigorous-intensity PA per day [27]. Yet, in order to improve all indices of physical fitness (muscle strength, motor fitness, and cardiorespiratory fitness) enhancing various PA is essential. Nevertheless, more research in larger preschool populations is needed in order to increase knowledge regarding these associations.

The primary strength of our study is the use of the DLW method which is considered the gold standard in measuring total energy expenditure in free-living settings [8]. The multipoint protocol was used to assess TEE via the DLW method. According to the International Atomic Energy Association (IAEA) handbook [32], the multipoint protocol is advantageous compared to the two-point method as data from several time points is collected which means data is averaged and the analytical error is reduced. Furthermore, Djafarian et al. [10] evaluated the two protocols in preschool-aged children and found that there were no significant differences between the average values for TEE between the two protocols. However, they stated that when maximum precision is desired the multipoint protocol should be used, such as for association studies such as this one [10]. The body composition indices used in this study were based on the 3-component model, which has been validated against the 4-component model and a very high agreement between the models has been found [35]. Furthermore, to our knowledge, there are no previous studies investigating the association of PAL and AEE (assessed using the DLW method) with physical fitness in preschool-aged children.

A limitation of this study is that we used a predicted BMR based on weight instead of a measured BMR. However, we also fitted PAL based on BMR predicted from fat and fat-free mass using raw data from previously published material in another sample of Swedish preschool-aged children [36] and the results were consistent (data not shown). The present study is also limited by the relatively small sample size (n = 40), which is common in studies using high cost methods (i.e., DLW). Hence, the findings should be confirmed using larger sample sizes. However, it is important to highlight that the children’s TEE was similar to data in children living in Western countries [37,38], and additionally, their body size was similar to Swedish reference data [39]. Finally, the level of maternal education level was higher than in the general Swedish population (79% versus 52%, respectively) [40]; however, when adjusting for maternal education in the models, conclusions remained the same.

## 5. Conclusions

In conclusion, a higher PAL and AEE at age 5.5 years were related to lower body fatness and improved lower-body muscular strength. Thus, enhancing energy expenditure in response to PA already at young ages may improve favorable body composition, and consequently, to prevent the development of obesity. Thus, future studies, using larger sample sizes and including more overweight and obese children as well as children from different socioeconomic backgrounds are needed.

## Figures and Tables

**Table 1:** Descriptive characteristics of the participating children (n = 40).

**Characteristics**	
Age, mean ± SD, years	5.6 ± 0.2
Height, mean ± SD, cm	114 ± 4.4
Weight, mean ± SD, kg	20.5 ± 4.2
BMI, median (range), kg/m^2^	15.1 (13.3–25.6)
**Body composition measurements**	
Total body fat, mean ± SD, %	25.1 ± 5.5
FMI, median (range), kg/m^2^	3.7 (2.14–11.8)
FFMI, median (range), kg/m^2^	11.3 (10.0–13.7)
**DLW measurements**	
TEE, mean ± SD, kJ/24 h	6040 ± 683
AEE, mean ± SD, kJ/24 h	2070 ± 487
BMR, mean ± SD, kJ/24 h	3970 ± 397
PAL, mean ± SD	1.52 ± 0.1
**Physical fitness tests**	
20-m shuttle run, mean ± SD, laps	8.58 ± 3.5
Handgrip strength, mean ± SD, kg	8.65 ± 2.2
Standing long jump, mean ± SD, cm	89.5 ± 16.8
4 x 10-m shuttle run, mean ± SD, s	16.2 ± 1.5

SD, standard deviation; BMI, body mass index; FMI, fat mass index; FFMI, fat-free mass index; DLW, doubly-labeled water; TEE, total energy expenditure; AEE, activity energy expenditure; BMR, basal metabolic rate; PAL, physical activity level.

**Table 2:** Associations of PAL and AEE with body composition (n = 40).

	PAL ^1^			AEE (kJ)/FFM (kg) ^2^		
	b (95% CI)	β	*p*-Value	b (95% CI)	β	*p*-Value
BMI (kg/m^2^)						
Unadjusted	−0.69 (−1.28 to −0.10)	−0.36	0.023	−0.04 (−0.06 to −0.01)	−0.45	0.004
Adjusted ^3^	−0.66 (−1.28 to −0.05)	−0.35	0.036	−0.04 (−0.06 to −0.01)	−0.44	0.006
Fat mass (%)						
Unadjusted	−2.29 (−3.64 to −0.94)	−0.49	0.001	−0.10 (−0.16 to −0.05)	−0.51	0.001
Adjusted ^3^	−1.95 (−3.24 to −0.66)	−0.41	0.004	−0.09 (−0.14 to −0.04)	−0.44	0.002
FMI (kg/m^2^)						
Unadjusted	−0.65 (−1.07 to −0.24)	−0.46	0.003	−0.03 (−0.05 to −0.01)	−0.51	0.001
Adjusted ^3^	−0.59 (−1.02 to −0.17)	−0.42	0.007	−0.03 (−0.05 to −0.01)	−0.47	0.002
FFMI (kg/m^2^)						
Unadjusted	−0.03 (−0.26 to 0.20)	−0.05	0.77	−0.01 (−0.02 to 0.00)	−0.20	0.22
Adjusted ^3^	−0.07 (−0.30 to 0.16)	−0.10	0.53	−0.01 (−0.02 to 0.00)	−0.25	0.11

PAL, physical activity level; AEE, activity energy expenditure; FFM, fat-free mass; CI, confidence interval; BMI, body mass index; FMI, fat mass index; FFMI, fat-free mass index. ^1^ The b coefficients provide estimates of the change in the body composition associated with a 0.1-unit change in the PAL. ^2^ The b coefficients provide estimates of the change in the body composition associated with a one-unit change in the AEE (kJ)/FFM (kg). ^3^ Adjusted for child’s sex and age at measurement.

**Table 3:** Associations of PAL and AEE with physical fitness (n = 40).

	PAL ^1^			AEE (kJ)/FFM (kg) ^2^		
	b (95% CI)	β	*p*-Value	b (95% CI)	β	*p*-Value
20-m shuttle run (laps)						
Unadjusted	0.62 (−0.34 to 1.58)	0.21	0.20	0.03 (−0.01 to 0.07)	0.24	0.15
Adjusted^3^	0.53 (−0.39 to 1.46)	0.18	0.25	0.03 (−0.01 to 0.07)	0.22	0.15
Handgrip strength (kg)						
Unadjusted	0.27 (−0.34 to 0.87)	0.14	0.38	0.00 (−0.03 to 0.03)	0.02	0.92
Adjusted ^3^	0.16 (−0.44 to 0.76)	0.09	0.59	−0.00 (−0.03 to 0.02)	-0.04	0.79
Standing long jump (cm)						
Unadjusted	6.04 (1.79 to 10.3)	0.42	0.007	0.26 (0.08 to 0.44)	0.43	0.006
Adjusted ^3^	5.28 (1.00 to 9.55)	0.37	0.017	0.23 (0.05 to 0.41)	0.38	0.014
4 × 10-m shuttle run (sec)						
Unadjusted	−0.33 (−0.72 to 0.06)	−0.27	0.094	−0.01 (−0.03 to 0.00)	−0.26	0.11
Adjusted ^3^	−0.26 (−0.65 to 0.13)	−0.21	0.19	−0.01 (−0.03 to 0.01)	−0.20	0.20

PAL, physical activity level; AEE, activity energy expenditure; FFM, fat-free mass; CI, confidence interval. ^1^ The b coefficients provide estimates of the change in the physical fitness associated with a 0.1-unit change in the PAL ^2^ The b coefficients provide estimates of the change in the physical fitness associated with a one-unit change in the AEE (kJ)/FFM (kg). ^3^ Adjusted for child’s sex and age at measurement.

## Supplementary Materials

The following are available online at http://www.mdpi.com/1010-660X/55/1/2/s1, Figure S1: Flow chart of the nested validation study in the MINISTOP trial.
