# Supplementary material for: Physical Activity Level Using Doubly-Labeled Water in Relation to Body Composition and Physical Fitness in Preschoolers
Source: Medicina (Kaunas). 2018 Dec 27;55(1):2. doi: 10.3390/medicina55010002 (PMC6359212; doi:10.3390/medicina55010002)
Supplement: Supplementary file 1 [file medicina-55-00002-s001.pdf]

Supplementary Figure 1. Flow chart of the nested validation study in the MINISTOP trial.

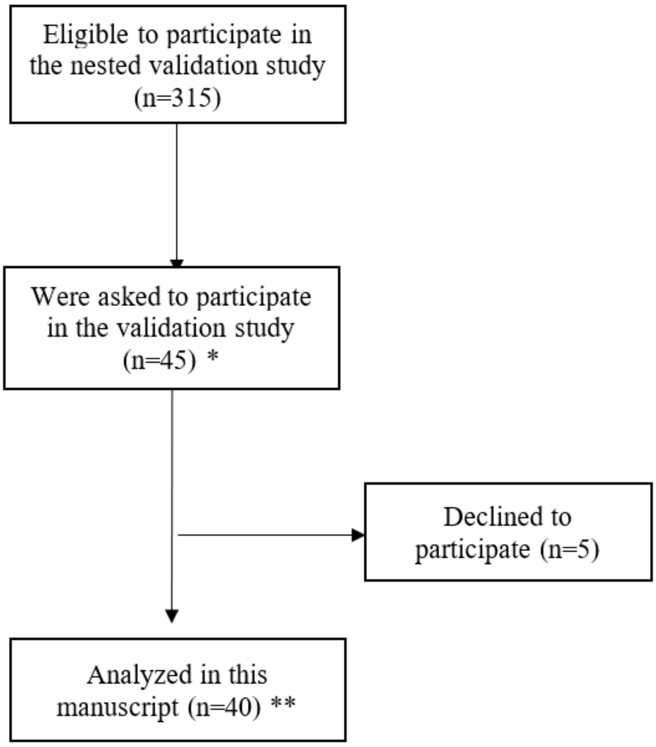

* As reported in the methods section, parents coming back for the follow-up measurement at 12 months after baseline were asked to participate in this nested validation study in the order they came back to the measurement. A total of 45 parents were asked to participate to get the required 40 parent and child dyads. **These parent and child dyads were not different with regards to baseline characteristics from the whole trial (n=315) [24,25].
